# Atomistic mechanisms for frictional energy dissipation during continuous sliding

**DOI:** 10.1038/s41598-021-99437-z

**Published:** 2021-10-07

**Authors:** S. Yu. Krylov, J. W. M. Frenken

**Affiliations:** 1 Institute of Physical Chemistry and Electrochemistry, Russian Academy of Sciences, Moscow, Russia 119071; 2 Advanced Research Center for Nanolithography, Amsterdam, 1098 XG The Netherlands

**Keywords:** Statistical physics, thermodynamics and nonlinear dynamics, Condensed-matter physics, Surfaces, interfaces and thin films, Nanoscience and technology

## Abstract

After more than a century of detailed investigations into sliding friction, we have not arrived yet at a basic understanding of energy dissipation, even for the simple geometry of a rigid slider moving over a perfectly periodic counter surface. In this article, we use a first-principles-based analysis to establish the atomistic mechanisms of frictional energy dissipation for a rigid object that moves continuously in the periodic surface potential landscape of a solid with vibrational degrees of freedom. We identify two mechanisms that can be viewed as (i) the continuous pumping of energy into the resonant modes, if these exist, and (ii) the destructive interference of the force contributions introduced by all excited phonon modes. These mechanisms act already in a purely dynamic system that includes independent, non-interacting phonon modes, and they manifest irreversibility as a kind of “dynamical stochastization”. In contrast to wide-spread views, we show that the transformation of mechanical energy into heat, that always takes place in real systems due to the coupling between phonon modes, can play only a minor role in the appearance of friction, if any. This insight into the microscopic mechanisms of energy dissipation opens a new, direct way towards true control over friction.

## Introduction

Friction is a phenomenon of high practical importance. In spite of the great progress made in the investigation of sliding friction and related phenomena^1–7^, our fundamental understanding of the origin of friction is still far from complete and a wealth of nontrivial physics remains hidden^8^. A thorough understanding of the fundamental mechanisms of energy dissipation will help us to gain true control over friction. By definition, friction is due to the irretrievable loss of the energy and momentum of the sliding body, and this loss is somehow related with the coupling to internal degrees of freedom. In the last decades, a large number of studies has been devoted to the possible roles of phononic, electronic and other excitations (see, e.g.^8^ and references therein) but the central question—why and how the exchange of mechanical energy from the moving slider to these internal degrees of freedom becomes irretrievable—remains to be answered. Unfortunately, this question is difficult to answer experimentally. At first glance, the obvious answer might seem that the irreversibility is directly associated with the transformation of mechanical energy into heat. This simple logic seems to be defied by results from straightforward atomistic modeling^9^, by the (unjustified) notion that excited phonons would “ballistically” carry energy away from the contact (see, e.g.^3^) and by recent, numerical calculations for a realistic phonon spectrum^10^, in which friction forces are found to arise even in the complete absence of damping, i.e. of thermalization of mechanical energy into heat. In a recent publication^10^, we demonstrated how the combined excitation of multiple phonons in an atomic-scale stick-slip event leads to motion that exhibits the characteristics of dissipation, even when these phonons are not damped. We now turn our attention to the more general question of how energy is dissipated and friction emerges during continuous sliding, when there are no slip events to excite the phonons simultaneously.

From a statistical-mechanics perspective^11^ and atomistic derivations of the Generalized Langevin Equation (see, e.g.^12^ and references therein and see below, in more detail), it can be argued that dissipative forces can be generated in a purely dynamic system of non-interacting phonon modes, where no thermalization can take place. As far as we know, in the framework of this first-principle-based approach, the concrete underlying mechanisms of frictional energy dissipation have never been addressed and neither has the possible role of thermalization that always takes place in real systems. In this paper we address these issues, following an atomistic derivation of the dissipative force and identifying the phononic mechanisms of sliding friction.

In the most familiar theoretical description of atomic-scale friction^1,8,13^, provided by the mechanistic Prandtl-Tomlinson model^14,15^, dissipation is not addressed explicitly. More sophisticated theories are usually based on the conventional Langevin equation (LE)

1 $$\begin{aligned} m{\mathop {\mathbf {r}}\limits ^{\cdot \cdot }} = - \nabla _{r} V(\mathbf {r})+\mathbf {F}_{\mathrm {diss}}+\mathbf {F}_{\mathrm {rand}} +\mathbf {F}_{\mathrm {ext}}. \end{aligned}$$

For the sliding motion of a body with mass *m* over a substrate with interaction potential $V(\mathbf {r}),$ this equation includes a dissipative force $\mathbf {F}_{\mathrm {diss}},$ a random force (thermal noise) $\mathbf {F}_{\mathrm {rand}}$ and a driving force $\mathbf {F}_{\mathrm {ext}}.$ The dissipative force is assumed to be proportional to the slider’s velocity $\mathbf {v}$ and a time- and velocity-independent damping factor (dissipation rate), $\eta .$

2 $$\begin{aligned} \mathbf {F}_{\mathrm {d}\mathrm {i}\mathrm {s}\mathrm {s}} = -\eta \mathbf {v}. \end{aligned}$$

In this form, the dissipative force is sometimes referred to as “viscous friction”. Although extremely helpful in describing many observations, ranging from macroscopic friction to refined experiments on the atomic scale with Friction Force Microscopy^1^, this approach remains semi-empirical: the value of $\eta$ is to be determined by a comparison of calculated forces with experiment, while the underlying dissipation mechanism remains hidden. Interestingly, one finds that stick-slip motion, as routinely observed on the full range of length scales, can be reproduced by the LE only if the value of the damping factor $\eta$ is constrained to a narrow interval, close to critical damping. We are only starting to find out what physics would restrict $\eta$ to such values^8,16^.

Within the context of non-equilibrium statistical mechanics^11,17^, the evolution of an object interacting with a bath of possible excitations is described by a Generalized Langevin Equation (GLE) with memory, which is expressed by a dissipative force of the following form,

3 $$\begin{aligned} \mathbf {F}_{\mathrm {diss}}(t) = -\int _{0}^{t}\widetilde{\mathbf {\eta }}(t -t^{ \prime })\mathbf {v}(t^{ \prime })dt^{ \prime } , \end{aligned}$$

with $\widetilde{\mathbf {\eta }}$ a memory kernel (generally, a tensor). According to Eq. (3), the force experienced at a given moment in time depends on how energy has been invested into and retrieved from the bath at previous times. The traditional model Eq. (2) tacitly assumes that the memory decay is extremely fast, ${\widetilde{\eta }}(t -t^{ \prime }) =\eta \delta (t -t^{ \prime }) ,$ but this does not find reasonable justification. In this perspective, friction is due to memory, rather than memory loss.

As was shown by a number of authors^12,18–20^ in the context of a variety of problems in condensed matter physics, for solid systems with vibrational degrees of freedom, the GLE can be derived from first-principles-based, atomistic considerations. Assuming independent, non-interacting phonon modes in the solid (and hence, the absence of any thermalization in the system) and a linear coupling between the motion of the rigid sliding object and the phonons in the substrate, the derivation (sometimes called “standard derivation”^21^) leads to a dissipative force of the form of Eq. (3), with the memory kernel $\widetilde{\mathbf {\eta }}$ explicitly related with the interatomic interactions. A remarkable feature of this approach is that frictional energy dissipation (at least the force proportional to velocity and directed against the motion) appears in a purely dynamical system without energy thermalization. If so, the phonons of a purely harmonic substrate act as the bath. It has been suggested^12,20^ that this could serve as the basis for a simulation method that would be alternative to the traditional Molecular Dynamics method and that would be free of the necessity to artificially introduce some kind of thermostat in the system.

As far as we know, besides a formal analogy with a “dynamical stochastization” similar to the Fermi–Pasta–Ulam problem^22,23^, little is known about how such a “dynamical bath” can work. Basically, at least three fundamental questions should be answered. First, under what circumstances is the “dissipative force” $\mathbf {F}_{\mathrm {diss}}$ really frictional and is the mean dissipation rate $\eta$ nonzero? Second, what are the physical mechanisms that lead to irretrievable loss of mechanical energy of the slider motion to the substrate? Third, will these dynamical mechanisms remain dominant in real systems where phonons always have a finite lifetime and thermalization, i.e. the transformation of mechanical energy into heat, does take place, after all.

**Figure 1:**
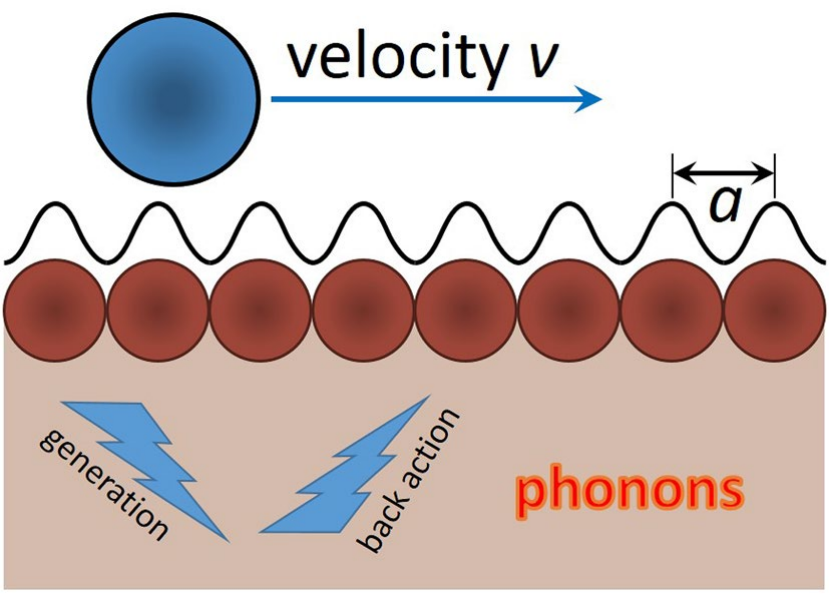
Schematic of the geometry considered in our theoretical treatment. A rigid slider, symbolized here as a single particle, is forced to travel at constant speed over a substrate, with which it experiences a perfectly periodic, sinusoidal interaction. This interaction leads to the excitation of phonons in the substrate, each exerting a force on the slider that oscillates in time. The total force, felt by the slider, is formed by the combination of all these oscillatory, phonon-based contributions. Even when no damping is introduced in this calculation, this combination exhibits the damping characteristics of a genuine friction force.

In this paper, we try to answer these questions following the formal results of the “standard derivation” and considering an (idealized but instructive) rigorously treatable problem of a particle moving with a given constant velocity in a periodic surface potential landscape (see Fig. 1).

We show, in particular, that a systematic transfer of mechanical energy of the relative motion of the particle into the phonon system of the periodic substrate is determined by two complementary mechanisms. They can be viewed as (i) the pumping up of “resonant” and “nearly-resonant” modes and (ii) the “destructive interference” of the force contributions related with all the other excited phonon modes. To our surprise, we find that, in combination, these two mechanisms lead (for a large solid) to a dissipative force that oscillates in time but has a nonzero, time-independent mean value. The oscillations of the dissipative force may seem unexpected but, actually, they are natural and they should not be confused with the trivial oscillations of the quasi-static (conservative) lateral force experienced by the slider. What is also interesting, is that the result is almost independent of the existence and value of the finite phonon lifetime. In other words, in the present case of continuous sliding, friction, a phenomenon of irretrievable loss of energy and momentum, is determined by these two dynamical mechanisms, and is independent of the subsequent transformation of the mechanical energy accumulated in the vibrational modes of the solid into heat.

## Dissipative force

Details of the atomistic derivation of the GLE can be found in the literature, see, e.g.^12,19,20^. In short, in the context of sliding friction, one starts with the full system of coupled Newtonian equations of motion for the sliding particle and for all atoms in the substrate. In the representation of normal vibrational modes of the substrate, their equations of motion turn out to be relatively simple, assuming a purely harmonic substrate and a linear coupling between the particle and motion of the substrate atoms. These two approximations guarantee that there is no direct or indirect coupling between the phonon modes, and that the interaction of the sliding particle with each of the phonon modes is independent of its interaction with the other modes. Then the phonon equations of motion can be solved analytically. Besides an obvious dependence on initial conditions, these solutions reveal the dependence of the current state of any mode on the entire history of the particle motion on the substrate. Substituting these solutions into the particle’s equation of motion, we arrive at GLE of the form of Eq. (1), with $V(\mathbf {r})$ the free energy. In addition to the quasi-static force, the equation includes the “dissipative” force of the form of Eq. (3) and a “noise” term that depends on the initial conditions. These three contributions constitute the full lateral force experienced by the particle. For a system that is in equilibrium at the start of the particle’s motion, the noise term constitutes a random force that satisfies the fluctuation-dissipation theorem. Within the scope of this paper, our interest is reduced to the dissipative force Eq. (3), which is independent of the initial conditions for the phonon modes, but depends on the initial conditions for the particle and the full history of its motion. The memory kernel has the form^12,19,20^

4 $$\begin{aligned} \widetilde{\mathbf {\eta }}[\mathbf {r}(t) ,\mathbf {r}(t^{ \prime }) ,t -t^{ \prime }] =\sum _{k}\omega _{k}^{ -2}\cos [\omega _{k}(t -t^{ \prime })] \nabla _{r}\phi _{k}[\mathbf {r}(t)] \nabla _{r}\phi _{k}^{ *}[\mathbf {r}(t^{ \prime })]. \end{aligned}$$

Here, $\mathbf {r}$ is the particle position, $\omega _{k}$ the frequency of the *k*-th phonon mode of the solid, and $\phi _{k}$ is the particle-phonon coupling coefficient for that mode, given by

5 $$\begin{aligned} \phi _{k} (\mathbf {r})=\left( \frac{ \partial W_{\mathrm {PS}}(\mathbf {r})}{ \partial q_{k}}\right) ^{(0)} , \end{aligned}$$

where $q_{k}$ stands for the generalized coordinate of the *k*-th mode and $W_{\mathrm {PS}}(\mathbf {r})$ is the potential energy of the particle–solid interaction calculated as a sum of all relevant atomic pair interactions, taking into account deviations of the solid atoms from their equilibrium positions; the superscript $\,^{(0)}$ indicates that the derivative is taken for equilibrium positions of all atoms in the lattice. As expressed in Eq. (5), $W_{\mathrm {PS}}$ and $\phi _{k}$ both depend sensitively on the position of the sliding particle, $\mathbf {r}$.

To clarify the origin of the dissipative force, it is convenient to rewrite the combination of Eqs. (3) and (4) as

6 $$\begin{aligned} \mathbf {F}_{\mathrm {diss}}(t) = -\sum _{k} \nabla _{r}\phi _{k}[\mathbf {r}(t)]\int _{0}^{t}\omega _{k}^{ -2}\cos [\omega _{k}\left( t -t^{ \prime }\right) ] \nabla _{r}\phi _{k}^{ *}[\mathbf {r}(t^{ \prime })]\mathbf {v}(t^{ \prime })dt^{ \prime } . \end{aligned}$$

Here, the time integral defines the generalized coordinate of the *k*-th phonon mode at the current time *t* as an integrated response of that mode to the particle’s motion over its entire trajectory from 0 to *t*. The structure of the integrand is straightforward. The *k*-th mode is excited at every point in time $t^{ \prime }$ with a contribution to its amplitude that is proportional to the particle velocity at that time and with an efficiency that is determined by the dynamic interaction gradient $\nabla _{r}\phi _{k}^{ *}[\mathbf {r}(t^{ \prime })].$ The phonon time correlation function $\cos [\omega _{k}\left( t -t^{ \prime }\right) ]$ in (6) describes the periodic evolution of that contribution between the time $t^{ \prime }$, at which it is generated, and the current time *t*. These contributions are integrated (without any form of damping), in order to accumulate the full history of the motion-induced excitation of the mode. The result is multiplied by $\nabla _{r}\phi _{k}[\mathbf {r}(t)] ,$ to get to the force experienced, in turn, by the sliding particle at time *t* due to excitation of mode *k* that it has generated itself between *t* and all the previous times $t^{ \prime } .$ The total dissipative force, acting on the particle is obtained as the sum over all phonon contributions.

The factor $\omega _{k}^{-2}$ appears in (4) and (6) in a natural way^12,19,20^ and it manifests the fact that the coupling of the slider motion to phonons is stronger for slower modes, which needs to be accounted for twice, namely both in the excitation of the phonons and in their delayed “back action” on the slider motion. Interestingly, this factor compensates the effect of the density of phonon modes that scales within the Debye model as $\omega _{k}^{2}$, thus introducing an essential influence on the magnitude of the dissipative force.

## Basic assumptions

We consider the idealized but instructive case that the sliding particle moves with a given constant velocity *v* (see Fig. 1). This situation is representative for the case that the particle is forced to slide by a hard spring. In a more general description, the velocity would not be constant, but it would evolve in accordance with the GLE. In our idealized case, we avoid such a variation and the dissipative force experienced by the particle is simply given by expression (6) with constant *v*. By dividing the dissipative force $\mathbf {F}_{\mathrm {diss}}(t)$ by $-v ,$ we obtain the dissipation rate $\eta .$ In our constant-velocity case, the dissipative force is reduced to the traditional form of (1), but with a time-dependent, microscopically defined $\eta (t).$

In order to capture the periodic nature of the particle–substrate interaction potential, with the substrate lattice period *a*,  we assume a sinusoidal form for all particle-phonon coupling factors $\phi _{k}(r) =A_{k}\sin (2\pi r/a +\alpha _{k} )$ and $r =vt ,$ so that

7 $$\begin{aligned} \nabla _{r}\phi _{k}(t) =A_{k}\frac{2\pi }{a}\cos \left( 2\pi \frac{v}{a}t +\alpha _{k} \right) , \end{aligned}$$

with the phases $\alpha _{k}$ determined by the position of the particle in the surface potential landscape at the starting point $t =0.$ Assumption (7) ignores the fact that, in principle, there can also be spatial periodicities at play, related with the wavelengths of the phonons. Effects related to these natural spatial periods can manifest themselves for a sufficiently small solid, when the phonons are “pinned” as standing waves to the geometry of the solid, e.g. with nodes at the edges. Such finite-size effects should not play a role for a large solid, for which such pinning does not occur as a result of the finite lifetimes of the phonons. Analysis of finite-size effects is in progress and will be reported elsewhere.

As a further, simplifying assumption, we take the amplitudes of the particle-phonon coupling factors $A_{k}$ equal for all modes *k*. Equal coupling of the particle to all phonon modes is probably far from realistic, but it should provide a meaningful first estimate in the limiting case of an atomically small contact between the particle and a single substrate surface atom. This is the only restrictive assumption of our approach, but we expect it not to be crucial for our results. Our aim is to identify the physical mechanisms responsible for a systematic, irretrievable loss of the slider’s mechanical energy to the substrate phonon system.

Summarizing, we follow the first-principle-based derivation of the dissipative force, and we explore the most general feature of the particle-solid interaction, namely its obvious lateral periodicity, with the lattice period of the substrate.

## Phononic mechanisms of sliding friction

We illustrate the result of our calculations by first examining the limit of a large solid, for which the phonon frequency spectrum tends to a continuous one. In this limit, the minimum phonon frequency approaches zero, while the maximum frequency $\omega _{\max }$ is always finite and reflects atomic-scale vibrations with the shortest wavelengths. In this continuum limit, we replace the summation over all phonon modes in Eq. (6) to an integration from 0 to $\omega _{\max } .$ In this integration, we assume a straightforward Debye density of phonon states that scales as $\omega _{k}^{2} ,$ appropriate for a three-dimensional solid. This allows one to correctly account for the full variety of phonon modes (for a given $\omega _{k}$ there are typically multiple modes with different wave vectors).

**Figure 2:**
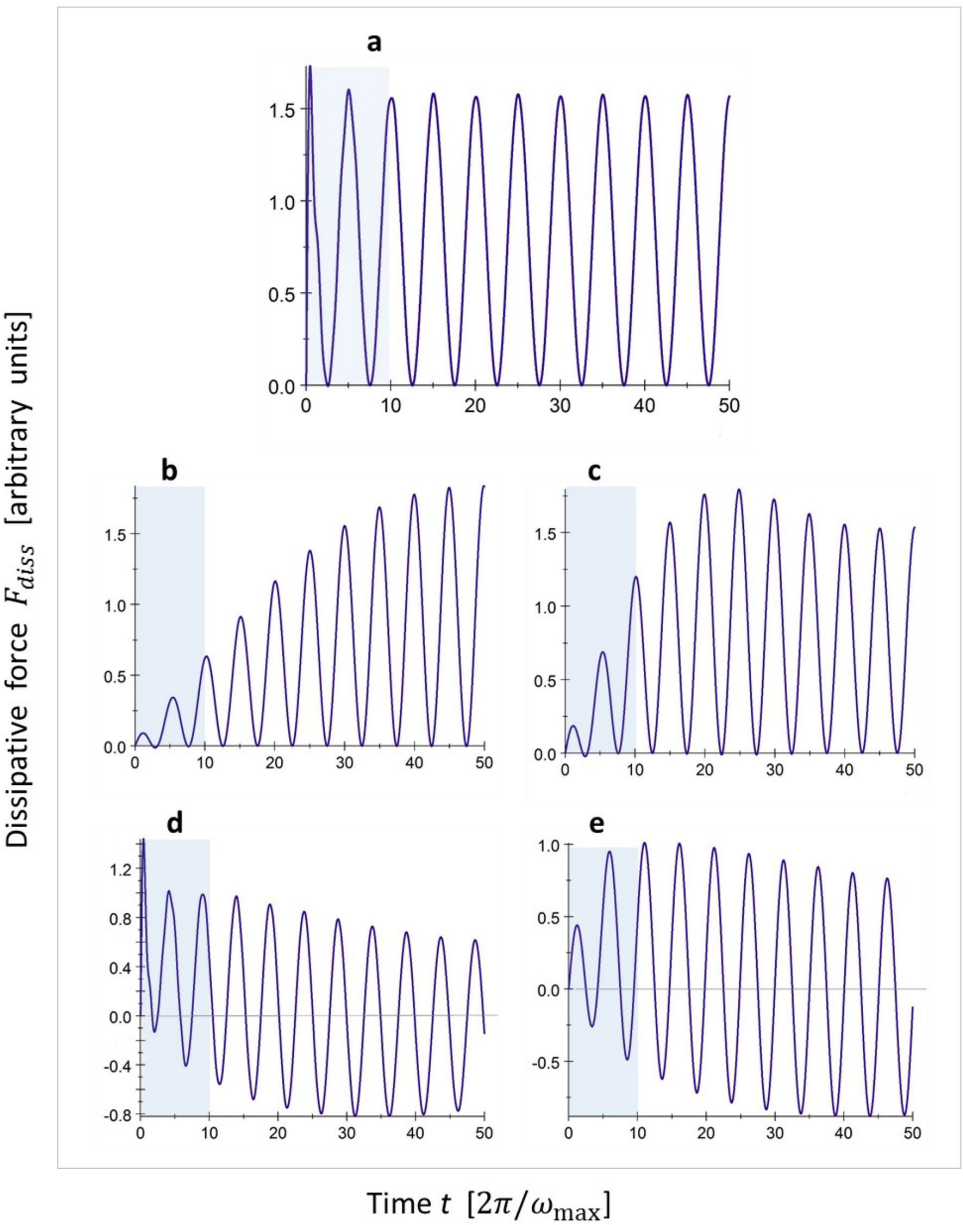
Dimensionless dissipative force (in arbitrary units) as a function of time (expressed in units of the shortest phonon period, $2\pi /\omega _{\mathrm {max}}$) for a particle sliding at constant velocity over an infinitely large, harmonic solid; positive values correspond to dissipation. In this example, the particle velocity is 10% of the velocity of sound, so that the washboard frequency is $0.10\,\omega _{\mathrm {max}}$. (**a**) Shows the result of integration over all phonon frequencies, from 0 to $\omega _{\mathrm {max}}$. The other panels correspond to contributions of particular phonon frequency ranges: a narrow range of frequencies within $\pm 10\%$ around the washboard frequency $2\pi v/a$ (**b**), a wider range of $\pm 20\%$ (**c**), and the upper (**d**) and lower (**e**) ranges complementary to the frequency range of (**c**). The first washboard period (the first lattice period passed by the particle) is marked in blue.

Results for this calculation are illustrated in Fig. 2, panel a.

Except for the very first peak at the start of the particle’s motion, the dissipative force exhibits very regular oscillations with double the “washboard” frequency $\omega _{\mathrm {w}\mathrm {b}} = 2\pi v/a$ that directly reflect the lattice periodicity (see (7)). The mean friction force is nonzero and constant in time and this remains unchanged, also for long times. In the example of Fig. 2, the particle velocity is chosen a factor 10 smaller than the sound velocity of the substrate. Changing velocity—even by an order of magnitude—we observe that the amplitude of the force oscillations (except for the first peak) and, hence, the mean friction force are linear in *v*,  in accordance with (2) and (6). This means that whereas the dissipation rate $\eta$ in equations of type (1) and (2) is velocity independent on average, on a more microscopic scale it is an oscillating function of time.

The result is practically independent of the starting point: the example of Fig. 2 is given for $\alpha _k= 0$ for all *k* in (7), but changing the initial phases causes only a change of the very first force peak and a certain phase shift of the force oscillations at longer times. Nevertheless, the amplitude of oscillations and the mean friction force turn out to be independent of initial phase. The starting point within one atomic spacing is not well defined in practice, and one can be interested to see whether and how the behavior is changed in the case of an ensemble average over the initial phases. Straightforward calculations show that this causes a certain (somewhat less than 40%) reduction of the amplitude of the force oscillations, with the instantaneous force values never reducing to zero, and, importantly, the mean friction force remains precisely the same as in the case of a single, fixed initial phase, such as the example shown in Fig. 2, panel a.

The observed oscillations of the dissipative force may seem surprising and they should not be confused with obvious inherent oscillations of the quasi-static lateral force given by the first term in r.h.s. of GLE (1). Space periodicity of the interaction, although hidden in the formal expression (3) for the dissipative force with memory, appears explicitly in the memory kernel (4). Interestingly, the dissipative force oscillations follow half of the lattice period (see Fig. 2) rather than the full one. The explanation is suggested by the structure of the general expression (6). Both the excitation of the phonon modes, as described by $\nabla _{r}\phi _{k}^{ *}[\mathbf {r}(t^{ \prime })],$ and the force response experienced by the particle, as determined by $\nabla _{r}\phi _{k}[\mathbf {r}(t)] ,$ oscillate with the washboard frequency, see (7). Consequently, the result is expected to have a $\cos ^{2}(\omega _{\mathrm {wb}}t)$ character. However the phonon time correlation function is not $\delta (t-t^{\prime })$ but $\cos (\omega _{k}(t-t^{\prime }))$, and the frequencies of the phonons involved are very different. In this perspective, the highly regular force oscillations of Fig. 2, panel a, which indeed appear to be close to $\cos ^{2}(\omega _{\mathrm {wb}}t),$ appear rather surprising.

Of course, the energy lost by the slider and accumulated in the substrate is equal to the integral of the dissipative force over the travelled distance. In our constant-velocity example this is directly proportional to a straightforward time integral of the dissipative force. For example, in the case of Fig. 2, panel a, the accumulated energy oscillates around a mean value that increases linearly in time.

To visualize the role of different phonons, in Fig. 2 we also present the force contributions of particular frequency ranges. Panels b and c show the contributions of two frequency windows centered around the washboard frequency, with bandwidths of ± 10% and ± 20% of $\omega _{\mathrm {wb}}$, respectively. For the narrower phonon range, panel b, one observes an increasing oscillation amplitude, which should be interpreted as a signature of the (nearly) resonant contributions, and the increase lasts up to relatively long times, after which the maximum force starts to saturate. For a wider phonon frequency window, panel c, the force rises more quickly and saturation appears earlier. With further widening of the range, the result rapidly approaches the total force, presented in panel a. Panels d and e show the effects of the frequency ranges that are complementary to that of panel c, namely with frequencies from 0 to $0.8\,\omega _{\mathrm {wb}}$ and from $1.2\,\omega _{\mathrm {wb}}$ to $\omega _{\mathrm {max}} ,$ respectively. The sum of the contributions in panels c–e is just what is shown in panel a. We see that phonons with frequencies that are not close to $\omega _{\mathrm {wb}}$ do not produce friction at long times but they do so at relatively short times and, importantly, they compensate the force increase related with nearly-resonant modes, which leads, as a result, to the regular oscillations of the total force and the time-independent mean friction force. In fact, we can see here a direct manifestation of dynamical stochastization. The coupling of the slider motion to each individual phonon mode oscillates with its own frequency and the corresponding “single-phonon memory” never decays. The associated force contributions are—in themselves—fully reversible (except for ‘the resonant” ones, see in detail below). Nevertheless, the integrated effect of many phonon modes turns out to be irreversible and the mean friction force is non-zero. The closeness of the force oscillations to $\cos ^{2}(\omega _{\mathrm {wb}}t)$, as simply prescribed by the velocity of the slider and the lattice periodicity, and the nearly complete absence of phonon signatures, manifests the rapid decay of the effective, integrated memory, which appears to be close to $\delta (t-t')$. The appearance of friction is directly related with this rapid decay of the combined memory. In a sense, this provides a partial justification why the conventional approach of Eq. (2) provides such a good approximation in many cases, albeit that we now recognize explicitly that on the microscopic scale the dissipation rate $\eta$ is time- and space-dependent.

**Figure 3:**
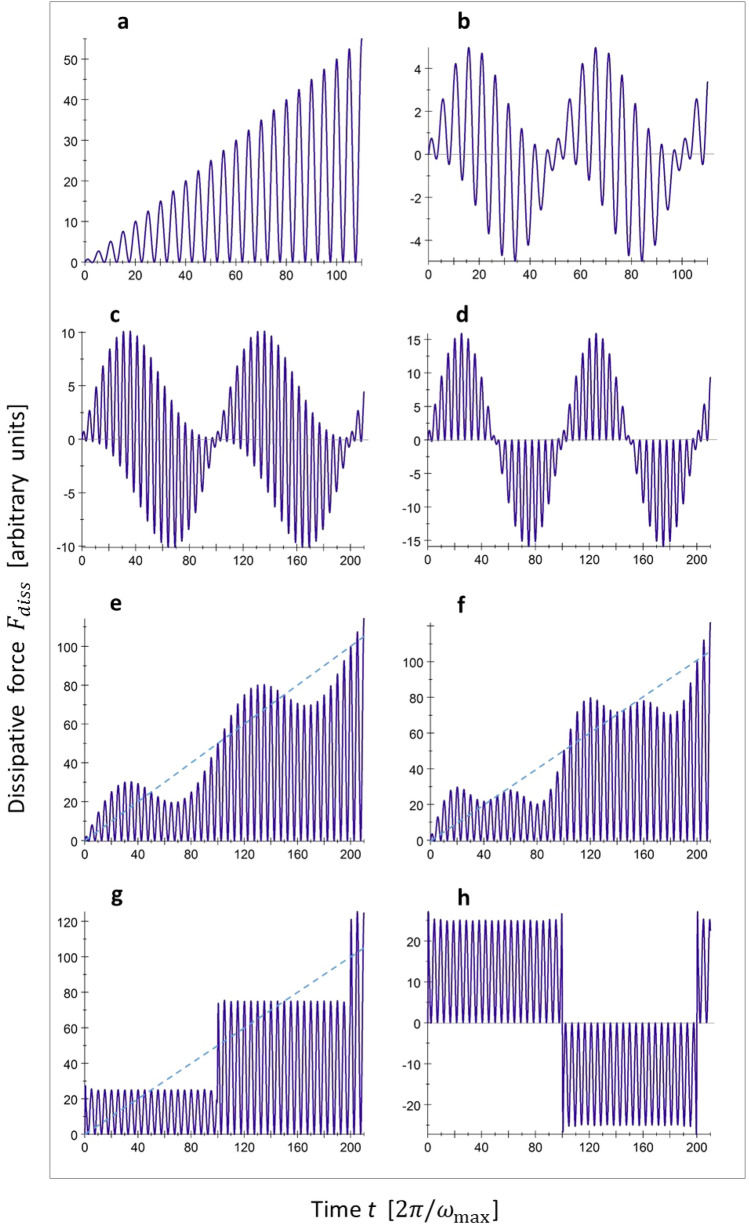
Single- and few-phonon contributions to the dissipative force as a function of time, calculated for a finite system with 100 equidistant phonon frequencies. In (**a–g**) the particle velocity is again set to 10% of the velocity of sound, so that the washboard frequency is $0.10\,\omega _{\mathrm {max}}$. (**a–c**) Demonstrate individual phonon contributions for specific frequencies: $0.10\,\omega _{\mathrm {max}}$ (resonance, (**a**)), $0.08\,\omega _{\mathrm {max}}$ (**b**) and $0.09\,\omega _{\mathrm {max}}$ (**c**). (**d–f**) Correspond to sums of several frequency contributions: $0.09\,\omega _{\mathrm {max}}$ and $0.11\,\omega _{\mathrm {max}}$ (**d**), $0.09\,\omega _{\mathrm {max}}$, $0.10\,\omega _{\mathrm {max}}$ and $0.11\,\omega _{\mathrm {max}}$ (**e**), $0.08\,\omega _{\mathrm {max}}$, $0.09\,\omega _{\mathrm {max}}$, $0.10\,\omega _{\mathrm {max}}$, $0.11\,\omega _{\mathrm {max}}$ and $0.12\,\omega _{\mathrm {max}}$ (**f**). As a continuation of this series, (**g**) shows the sum over all 100 frequency contributions (which formally correspond to the spectrum of a Debye solid of $100\times 100\times 100$ atoms). (**h**) Is similar to (**g**), but with the washboard frequency shifted away from resonance to $2\pi v/a=0.105\,\omega _{\mathrm {max}}.$ Blue dotted lines are guides to the eye and indicate the growing force amplitude of the resonant contribution, seen in (**a**).

In order to see how this compensation effect works, it is instructive to inspect individual contributions to $\mathbf {F}_{\mathrm {d}\mathrm {i}\mathrm {s}\mathrm {s}}(t) ,$ related with specific phonon modes. In particular, we should distinguish two classes of modes, namely those with a frequency $\omega _{k}$ that coincides precisely with the velocity-dependent washboard frequency $\omega _{\mathrm {w}\mathrm {b}} = 2\pi v/a$ and those for which this is not the case. The former class can be considered “resonant” with the periodic excitation, introduced by the sliding particle. Resonant and non-resonant contributions to $\mathbf {F}_{\mathrm {d}\mathrm {i}\mathrm {s}\mathrm {s}}(t),$ obtained from a calculation for a finite system with 100 equidistant phonon modes are shown in Fig. 3, panels a–c.

In the resonant case, the oscillation amplitude and the mean force both increase linearly in time (panel a). This linear increase may seem counterintuitive, but it is completely in line with the general mechanics of driven vibrational motion in the absence of damping. When an oscillator is subjected to a resonant, periodic excitation of constant strength, its amplitude increases linearly in time, and so does the force response experienced by the driver. Clearly, the resonant modes of the substrate are permanently pumped up in this way and the corresponding energy is lost by the sliding particle.

Each of the non-resonant contributions (Fig. 3, panels b and c) exhibits a complex behavior that combines oscillations with twice the washboard frequency, as an effect of the lattice periodicity, and “super-oscillations” reflecting the effect of memory: the investment of the particle’s energy into the substrate’s mode is periodically changed into the return of energy from the mode to the particle. The period of the super-oscillations of $2\pi /\mid \omega _{k}-\omega _ {\mathrm {wb}}\mid$ is completely determined by the frequency difference. On a large time scale, the non-resonant contributions oscillate symmetrically around zero, with a zero mean value. The energy exchange between the particle and each non-resonant mode is periodic, without leading to a systematic transfer of energy from the particle to the substrate or vice versa. This may make most of the modes seem irrelevant for friction in the system of independent phonons, because of their “wrong” frequency, but this view is not justified. Panel d shows the combined effect of two modes with different frequencies, namely one below the washboard frequency ($\omega =0.9\,\omega _{\mathrm {wb}} ;$ see panel c) and one above, $\omega =1.1\,\omega _{\mathrm {wb}}.$ We see that the interference of the two modes causes a more rapid increase of the force oscillations than in the resonant case, as well as changes in the sign of the force that are different with respect to single-phonon case. As a result, the combined result of these two modes plus the resonant one exhibits a stair-case-type behavior, see Fig. 3, panel e. This is in contrast to the simple, linear increase of the resonant contribution, indicated in panel e by the blue dotted line. With the addition of further non-resonant modes, the staircase becomes more pronounced, panel f. When a large number of non-resonant modes are taken into account, panel g, the stairs are close to ideally flat. The explanation for this behavior is straightforward. Each of the non-resonant modes starts with a positive contribution at $t=0$ (panels b and c in Fig. 3), and so does the sum of the contributions of all non-resonant modes. The time evolution of this sum is characterized by “destructive interference” between the (non-resonant) modes, which actually makes the sum decrease at later times, almost perfectly compensating the continued growth of the resonant contribution. The interference leads to the counterintuitive result that even though the sliding particle periodically invests and recollects equal amounts of energy into and from each non-resonant mode, the sum of all these exchanges exhibits a long-term signature.

The time duration of the first plateau reflects a finite-size effect that is completely determined by the difference between the discrete phonon frequency levels. In the example of Fig. 3, panel g, that difference is $0.01\,\omega _{\mathrm {max}},$ so that the plateau has a length of $100/\omega _{\mathrm {max}}.$ With increasing size of the solid and, hence, decreasing frequency difference, the time duration of the first plateau grows up to infinity, in full accordance with the long-term behavior of the dissipative force in the continuum limit, presented in Fig. 2, panel a.

Interestingly, if the particle velocity is such that there is no exact resonance, the first force “plateau” remains practically the same, as is illustrated by Fig. 3, panel h for a wash board frequency slightly above $0.10\,\omega _{\mathrm {max}},$ but instead of a subsequent step-like increase, the force changes sign, exhibiting a super-oscillation for the full sum of forces and a corresponding mean friction force that tends to zero on a large time scale. This suggests that for a sufficiently small solid with a pronouncedly discrete phonon spectrum, the friction behavior can be very different, depending on whether or not the resonance conditions are met. Again, this is a finite-size effect. For an infinitely large solid, for which the first plateau is infinitely long, such a difference is rendered irrelevant.

## On the role of thermalization

**Figure 4:**
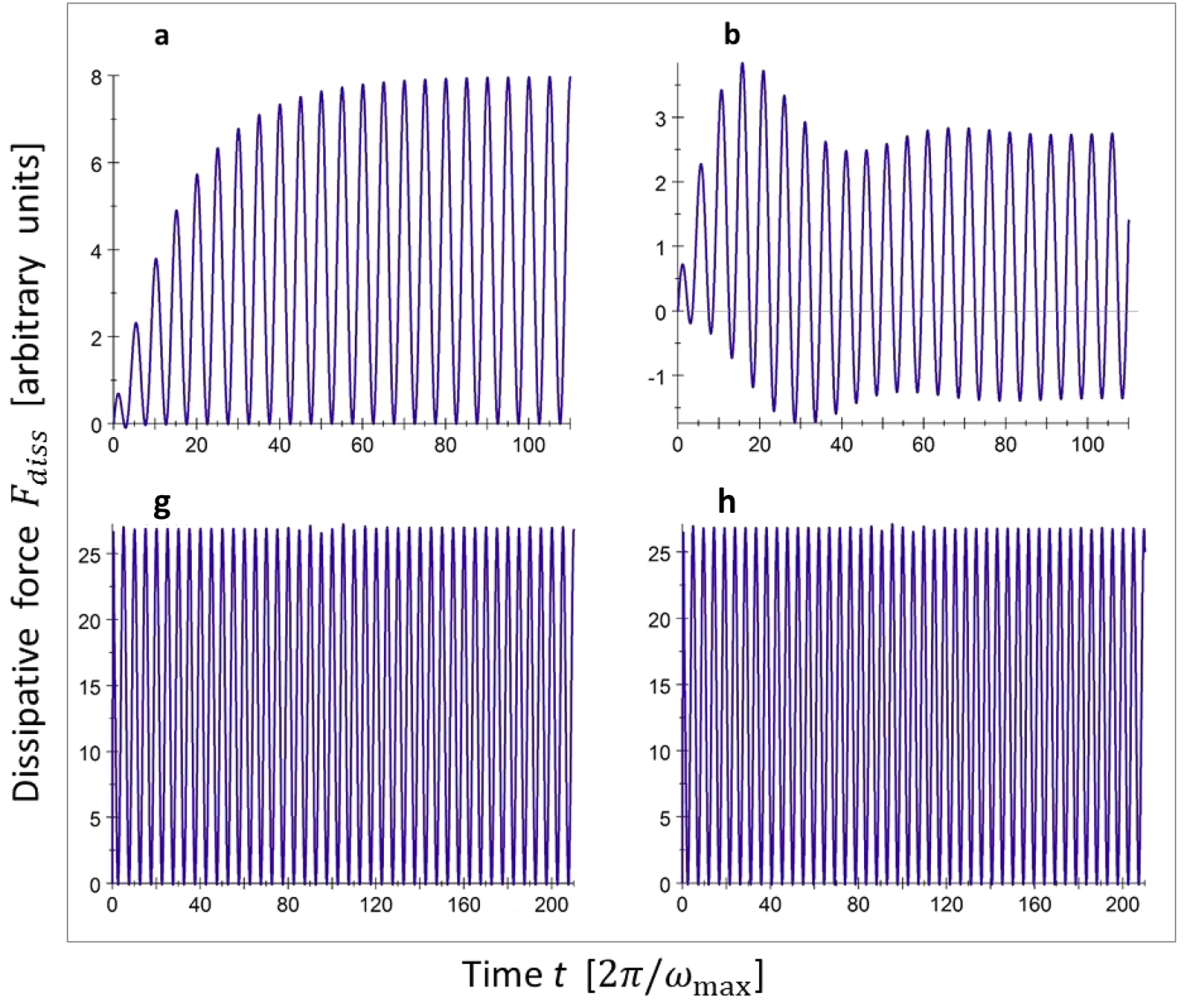
Similar to Fig. 3, but taking into account finite lifetimes of all phonons of 10 vibrational periods, $\Gamma = 10$ (see text). The labels refer to the corresponding panels of Fig. 3.

At this point, we turn to what is perhaps the most relevant question, which is whether these mechanisms of irretrievable energy loss, namely, the continuous feeding of energy to the (nearly)-resonant phonon modes and the destructive interference of all the others, are also dominant in real solids. The crudest assumption that we need to reconsider is that of the complete absence of damping. In reality, phonon lifetimes are always finite, due to anharmonicity of the atomic interactions in the solid and as a result of crystal lattice imperfections. In due time, this will always redistribute the sliding-induced phonon excitations over all vibrational degrees of freedom and re-establish an equilibrium distribution of phonon occupation levels. Surely, this redistribution will erase all memory and render the lost energy of the sliding particle truly irretrievable. This memory loss has been ignored completely in the derivation of (6). In order to estimate the role of this thermalization of the lost energy, we introduce the finite lifetime of phonons in our calculations as an exponential memory decay through an additional factor $\exp \left( -\frac{t -t^{ \prime }}{\tau _{k}}\right)$ in the time integral in (6). For simplicity, we assume that for each phonon mode *k*, the lifetime $\tau _{k}$ is a factor $\Gamma$ larger than the phonon period, i.e. $\tau _{k} =\Gamma \frac{2\pi }{\omega _{k}} ,$ so that each phonon ‘lives’ for an average of $\Gamma$ periods. In Fig. 4 we adopt an extreme level of damping of $\Gamma = 10$ periods and again inspect selected single-phonon contributions.

Due to the finite phonon lifetimes, the force contributed by the resonant mode (panel a) no longer diverges but instead grows towards a maximum level that is proportional to $\Gamma$. For the non-resonant modes we recognize two effects in Fig. 4, panel b. First, the long-time average of the force experienced by the particle due to the non-resonant modes becomes nonzero. For modes not too close to resonance, this contribution is proportional to $\Gamma ^{-1},$ which can be regarded as a manifestation of the irretrievable loss of mechanical energy due to the transformation into heat. Second, the super-oscillations characteristic for non-resonant modes with infinite lifetimes (compare with Fig. 3, panel b), are damped. We find that the reduction in force contributed by the resonant and near-resonant modes is largely cancelled by the appearance of nonzero contributions from the other, non-resonant modes. This cancellation makes the overall influence of phonon thermalization on the dissipative force surprisingly weak. Even for the very severe phonon damping of $\Gamma =10,$ the force oscillation amplitudes and the mean friction force turn out to change less than $10\%,$ as seen in Fig. 4, panels g and h. Note, that the damping has removed the long-term staircase and up-and-down behavior that were present as finite-size effects in Fig. 3, panels g and h. Results similar to those in Fig. 4 for a discrete phonon spectrum are obtained from calculations in which we integrate over the full, continuous phonon spectrum of a large solid with damping. The finite phonon lifetimes hardly produce any changes with respect to the dissipative forces in Fig. 2, panel a for the completely undamped situation. We see in all these cases that even in the presence of strong phonon damping, friction emerges in a natural way as an intrinsic memory effect and the direct transformation of mechanical energy into heat is not essential.

## Summary

In conclusion, in answer to the question where friction comes from, we distinguish three basic mechanisms of irretrievable loss of mechanical energy and momentum. These are (i) the continuous pumping of the phonon modes that are resonant or nearly resonant with the velocity-dependent washboard frequency of the sliding motion; (ii) the destructive interference between the contributions of all phonon modes of the solid; (iii) direct transformation of mechanical energy of excited phonon modes into heat. Mechanisms (i) and (ii) work at long and short times, respectively. The interplay between different vibrational modes is intricate and leads to enormous variations in time of the relative contributions of (near)-resonant and other modes. When combined, mechanisms (i) and (ii) produce a constant mean friction force, almost immediately after the slider starts moving. This is so even for a perfect, harmonic solid with infinite phonon lifetimes, in which mechanism (iii) is not active. In realistic solids, phonon lifetimes are always finite and mechanism (iii) comes into play, reducing the dominant role of the resonant modes and increasing the bandwidth around resonance of modes noticeably contributing to friction. But the influence of mechanism (iii) on the total friction force is practically irrelevant.

## Outlook

The observed, perfect interplay between resonant and non-resonant modes is surprising and may be a direct consequence of our simplifying assumptions of a phonon spectrum with equidistant frequencies and of equal coupling strength between the moving particle and each of the phonon modes. In the context of this article, we have not explored whether more realistic choices for the spectrum and the coupling would lead to a definite time signature in the total friction force.

Even though our analysis is formulated in the context of phononic excitations, the basic concept is more general and applies just as well to other internal degrees of freedom of the solid. The restriction to a constant sliding velocity should also not be regarded as a limiting aspect. If the velocity changes in time, other phonons will be resonant with the slider’s new washboard frequency, while the destructive interference will always be active.

In this paper we only lightly touched upon finite-size effects. These will be considered more extensively elsewhere. However, one important prediction can be made already at this stage. If the washboard frequency is well outside the frequency ranges covered by the system’s phonon bands, mechanisms (i) and (ii) cannot contribute to the mean friction force (see Fig. 2, panel d) and, hence, only the phonon thermalization mechanism (iii) remains. Consequently, for sufficiently small substrate dimensions and low slider velocities, friction should be substantially reduced. This can serve as the possible, first-principles-based explanation of the reduction of friction on sub-micron surface islands, that was recently observed experimentally^3^, and it illustrates how the new insights on the microscopic mechanisms of energy dissipation, presented in this article suggest new, direct ways towards true control over friction.

### Human participants or animals

This research does not involve Human Participants and/or Animals.

## Data Availability

No datasets were generated or analysed during the current study.
